# Effects of global change factors and living roots on root litter decomposition in a Qinghai-Tibet alpine meadow

**DOI:** 10.1038/s41598-019-53450-5

**Published:** 2019-11-15

**Authors:** Meng Shu, Qingzhou Zhao, Zhen Li, Lin Zhang, Peng Wang, Shuijin Hu

**Affiliations:** 1 Colloge of Resources and Environmental Sciences, Nanjing Agricultural University, Nanjing, Jiangsu 210095 China; 2 Department of Entomology & Plant Pathology, North Carolina State University, Raleigh, NC 27695 USA

**Keywords:** Climate-change ecology, Grassland ecology

## Abstract

Roots account for a major part of plant biomass in Tibetan alpine meadows. Understanding root decomposition with global change is key to predict carbon (C) and nutrient dynamics on the Qinghai-Tibet Plateau. Yet, few experiments have carefully examined root decomposition as influenced by global change. We conducted a field study to investigate the effects of nitrogen (N) addition, air warming, precipitation change, and the presence/absence of living roots on root decomposition in a Tibetan alpine meadow. Our results showed that N addition increased the mass and C remaining, and induced N accumulation in the litter. Increased precipitation significantly amplified the positive effect of N addition on litter mass remaining. The presence of alive roots in the litterbags decreased root litter C remaining but significantly increased N and phosphorus remaining of the litter. However, we did not find any significant effects of air warming on the litter decomposition. In the Qinghai-Tibet Plateau, N deposition is predicted to increase and precipitation regime is predicted to change. Our results suggest that the interaction between increased N and precipitation may reduce root decomposition in the Qinghai-Tibet Plateau in the future, and that the large stock of living roots exert a dominant impact on nutrient dynamics of root decomposition in the Tibetan alpine systems.

## Introduction

Anthropogenic activities have been altering multiple environmental factors including temperature, precipitation, and N deposition^1,2^. The Qinghai-Tibet Plateau is especially sensitive to human disturbance and global change factors^3^. Warming and increasing precipitation in the Qinghai-Tibet Plateau is a pivotal issue^4,5^, which may significantly affect soil microbes and their decomposition activities. Decomposition of plant litter plays an important role in carbon (C) and nutrient cycling in terrestrial ecosystems^6,7^, as it regulates the recycling of nutrients from litter materials^8^, controls the C efflux from the soil and influences the formation and stabilization of soil organic matter^9,10^. The soil in the Qinghai-Tibet Plateau contains 34 Pg C and constitutes an important C pool in China’s terrestrial ecosystems^11^. In addition, alpine meadows of the Qinghai-Tibet Plateau have high root biomass^12,13^ which constitutes the dominant source of soil organic carbon^14^, making root decomposition particularly critical in C flux and nutrient cycling in these ecosystems. Therefore, changes in root decomposition caused by global change can have large consequences on C and nutrient stocks in the Qinghai-Tibet Plateau.

Litter decomposition, including root decomposition, is strongly affected by abiotic factors such as temperature, moisture and nitrogen (N) availability. Many studies have showed that climate warming can stimulate litter decomposition^15,16^, especially in high-latitude and high-altitude areas where litter decomposition is limited by low temperature^17,18^. Precipitation may affect litter decomposition by changing soil water availability and thus microbial activities^19,20^. Increasing N deposition can also influence litter decomposition through changing microbial activities^21,22^. However, inconsistent results were reported on the effects of N addition on litter decomposition^23,24^, and the effects of N addition on root decomposition are still unclear.

Beside abiotic factors, root litter decomposition is also under the influence of living roots in the nature^25^. On the one hand, living roots can enhance the decomposition of litter through the priming effects of their labile root exudates which stimulate microbial enzyme production^26^. On the other hand, roots can suppress litter decomposition by outcompeting microbes for soil moisture and nutrients or exuding labile organic compounds that microbes prefer to use over litter and thus suppress microbial production of enzymes for decomposition^27,28^. However, the net effect of living roots on root litter decomposition is still unclear, particularly in a scenario with multiple changing factors such as water, temperature and N availability, all of which can influence the growth of roots and microbes as well as their interaction.

The large proportion of roots in alpine meadows in the Qinghai-Tibet Plateau makes effects of living roots on the decomposition of dead roots particularly critical in C flux and nutrient cycling in these ecosystems. However, previous studies in the Qinghai-Tibet Plateau have mainly focused on the decomposition of aboveground litter. In order to better understand C and nutrient cycling in the belowground part of the Qinghai-Tibet Plateau, we investigated the effects of global change factors, including temperature, precipitation and N availability, and the presence/absence of living roots on early stage of root decomposition in an alpine meadow of the Qinghai-Tibet Plateau.

## Results

### Treatment effects on soil inorganic N, soil temperature and soil moisture

**Figure 1:**
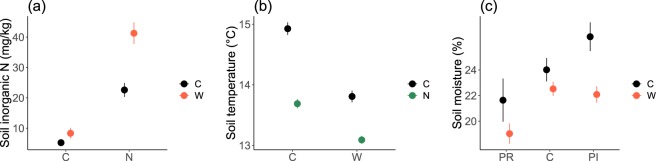
Soil temperature (**a**), soil moisture (**b**) and soil inorganic N (**c**) in different treatments. C, control; W, air warming; N, nitrogen addition; PI, precipitation increase; PR, precipitation reduction. Soil temperature and soil inorganic values were averaged across the three precipitation treatments (*n* = 6 and *n* = 12, respectively), soil moisture values were averaged across the two N treatments (*n* = 4). Error bars denote 1 standard error. See Table S1 for the means and standard errors of all the 12 treatments and Table S2 for the full model with all the main effects and their interactions.

Soil inorganic N increased with N addition and air warming (6.8 ± 0.1 *vs* 32.0 ± 0.1 and 13.9 ± 0.1 *vs* 24.8 ± 0.1 mg/kg, respectively; *F*_1,33_ = 66.7, *p* < 0.001 and *F*_1,33_ = 12.5, *p* = 0.001, respectively; Fig. 1a; Tables S1 and S2), and it was increased even more by the combination of two treatments (*p* = 0.016). Soil temperature was significantly decreased by the treatment of N addition and air warming (14.37 ± 0.14 °C *vs* 13.39 ± 0.08 °C and 14.31 ± 0.14 °C *vs* 13.45 ± 0.09 °C; *F*_1,12_ = 52.1, *p* < 0.001 and *F*_1,12_ = 40.1, *p* < 0.001, respectively; Fig. 1b). Soil moisture was significantly higher in the increased precipitation treatment than in the reduced precipitation treatment (24.3 ± 0.9% *vs* 20.3 ± 1.0%; *F*_2,11_ = 4.1, *p* = 0.046; Fig. 1c); soil moisture was also higher in the non-warming treatment than in the air warming treatment (21.2 ± 0.5% *vs* 24.1 ± 0.8%, *F*_2,11_ = 5.9, *p* = 0.033; Fig. 1c).

### Effects of air warming, N addition, precipitation changes and living roots on litter mass, C, N and P remaining

**Figure 2:**
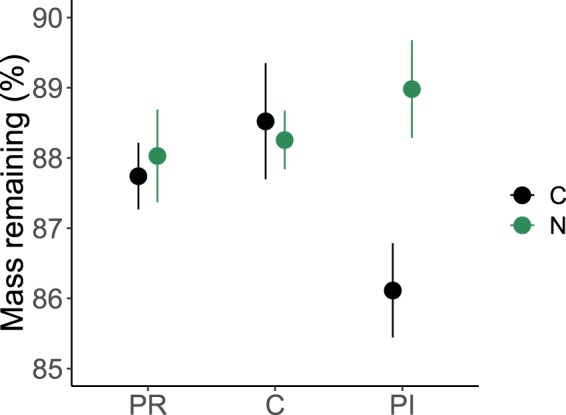
Mass remaining of the litter of mixed roots from dominant species on the Qinghai-Tibet Plateau in different treatments and the effects of N addition and precipitation change. C, control; N, nitrogen addition; PR, precipitation reduction; PI, precipitation increase. All data were expressed as % of original litter mass. Bars show mean ± SE (*n* = 8). See Table S3 for the full model with all the main effects and their interactions.

Litter decomposition was in general very slow in our study, and on average about ninety percent (87.9 ± 1.3%) of initial mass remained after the growing season (Figs 2 and S2). None of the measured litter properties was significantly affected by the air warming treatment (Figs S1 and S2). Litter mass remaining was marginally affected by N addition (*F*_1,33_ = 3.9, *p* = 0.055; Table S3) and was significantly affected by the interaction of N and precipitation (*F*_2,33_ = 3.6, *p* = 0.039; Table S3): mass remaining in the treatment of both N addition and precipitation increase was higher than precipitation increase alone (89.1 ± 0.7% *vs* 85.6 ± 0.7%).

**Figure 3:**
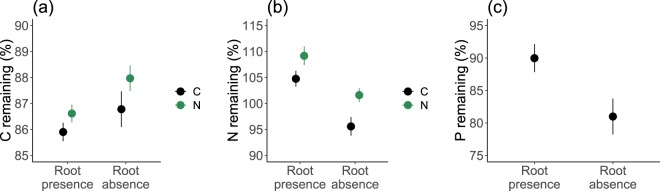
C (**a**), N (**b**) and P (**c**) remaining of the litter of mixed roots from dominant species on the Qinghai-Tibet Plateau and the effects of root absence/presence and N addition. C, control; N, nitrogen addition. All data were expressed as % of original litter C, N and P content. Bars show mean ± SE (*n* = 12 for C and N remaining and *n* = 24 for P remaining).

Initial C, N, and P concentrations of the root litter were 47.4%, 0.4%, and 0.07%, respectively. During the four months of decomposition, C and P contents in the litter were reduced across all the treatments (Figs 3a,c and S2). N did not show significant reduction, on the contrary, it even showed net accumulation when N was added or when living roots were present (Figs 3b and S2). C remaining ranged from 84.6% to 89.1% across all treatments, and N, P remaining ranges from 88.5% to 112.6% and from 66.3% to 98%, respectively. C remaining was marginally higher with N addition than without N addition (*F*_1,33_ = 4.0, *p* = 0.053). Besides, C remaining and N remaining were marginally higher when both N and precipitation were added than when precipitation was added alone (87.9 ± 0.5% *vs* 85.3 ± 0.5%, 106.1 ± 1.4% *vs* 96.5 ± 2.0% for C and N remaining, respectively; Table S3). Moreover, N remaining was significantly increased by N addition (*F*_1,68_ = 9.8, *p* = 0.003; Table S3) compared to ambient N condition (105.4 ± 1.2% *vs* 99.9 ± 1.3%).

C, N and P remaining were all significantly affected by the presence/absence of living roots (*F*_1,36_ = 5.4, *F*_1,68_ = 25.6 and *F*_1,35_ = 7.8, *p* = 0.026, < 0.001 and = 0.008 for C, N, and P remaining, respectively; Fig. 3; Table S3). C remaining in the presence of living roots was 86.3 ± 0.2%, which was less than that in the absence of living roots by 1.08%. Both N remaining and P remaining in the presence of living roots were higher than that in the absence of living roots, by 8.09% and 8.99%, respectively.

### Effects of living roots on litter nutrient stoichiometry (C:N and C:P ratios)

**Figure 4:**
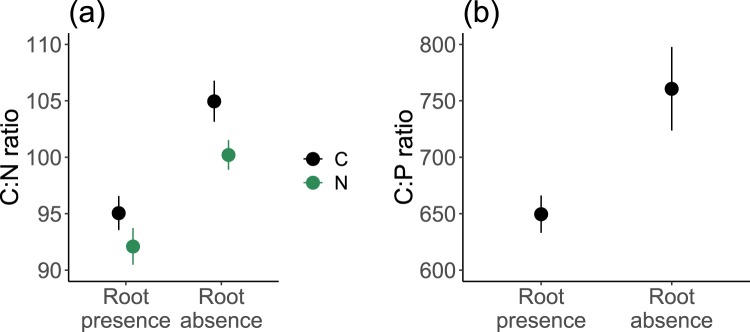
C:N (**a**) and C:P ratio (**b**) of the litter of mixed roots from dominant species on the Qinghai-Tibet Plateau and the effects of root absence/presence and N addition. C, control; N, nitrogen addition. Bars show mean ± SE (*n* = 12 for C:N ratio and *n* = 24 for C:P ratio).

The initial C:N of the root litter mixture was 116.9. C:N ratios in the decomposed litter (ranging from 88 ± 4.3 to 110.7 ± 4.1) were lower than the initial C:N ratios in all treatments. In addition, there was a significant reduction in C:N ratio when living roots were present compared to that when living roots were absent (93.6 ± 1.1 *vs* 102.6 ± 1.2, *p* < 0.001; Fig. 4; Table S3).

The initial C:P ratio of the root litter mixture was 685.4. C:P ratios of the decomposed litter were in general lower than the initial C:P ratios in the presence of living roots, while the changes in C:P ratio between decomposed litter and initial litter in the absence of living roots were inconsistent. There was a significant reduction in C:P ratio when living roots were present compared to that when living roots were absent (649.6 ± 16.5 *vs* 760.7 ± 37, *p* = 0.006; Fig. 4; Table S3).

The initial N:P ratio of the root litter mixture was 5.8, and after the decomposition N:P ratio of the litter increased to 7.2, as a result of accumulated N and decreased P concentration. However, we did not find any significant differences in N:P ratio between the treatments.

### Relationships between litter parameters and soil parameters

**Figure 5:**
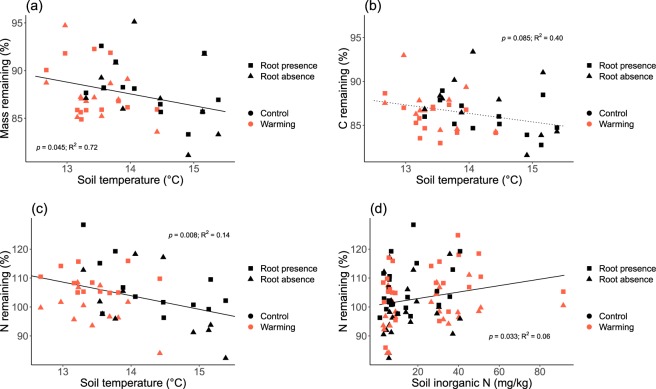
Significant relationship between measured soil variables and decomposition aspects. Square points in rectangular denote litterbags with living roots present, triangular points denote litterbags with living roots absent; points in black denote litterbags in the plots without air warming, points in red denote litterbags in the plots with air warming of OTC. Solid lines denote significant relationships, and dashed line denote marginally significant relationship.

The mass remaining and N remaining were significantly correlated with soil temperature and decreased with increasing soil temperature (Fig. 5a,c). The relationship between C remaining and soil temperature was marginally significant (Fig. 5b). N remaining was also significantly correlated with soil inorganic N and increased with increasing soil inorganic N (Fig. 5d). The variance in the N remaining explained by soil temperature was larger than that explained by soil inorganic N (R^2^ = 0.014 and 0.06, respectively; Fig. 5c,d).

## Discussion

The Qinghai-Tibet Plateau has high root biomass, and the decomposition of root litter can have large influence on ecosystem C fluxes. While global change can greatly change leaf litter decomposition, its effects on root decomposition in the Qinghai-Tibet Plateau are not well studied yet. In this study we investigated the effects of three global change factors, including temperature, precipitation alteration and N deposition, as well as the net effect of living roots on root decomposition. We found that air warming had insignificant effects on root decomposition, while N addition and precipitation alteration, particularly precipitation increase can have large influences on root decomposition. Besides, we found that living roots played a dominant role in the nutrient dynamics of root decomposition.

In our study, N addition showed marginal positive effects on root litter mass remaining and C remaining (Figs 2 and 3a). Many other studies have shown that mass remaining of litter was higher with N addition, and it is often related to decreased lignin-degrading enzyme activities^29–31^. Our results also showed that N addition significantly increased total N in the decomposing litter (Fig. 3b), and remaining N increased with soil N concentration (Fig. 5d). There can be two reasons. First, the C:N ratio of our initial root litter was pretty high (116.9), and soil microbes, particularly fungi, may translocate N from the outside of litterbags^32,33^, and this translocation may be greater with higher N availability under N addition. However, we could not examine this hypothesis as we did not measure soil microbes and their activities in this study. Second, it is possible that some applied N was chemically fixed due to the improved N availability, as exogenous N can bind with substrates such as lignin in the litter^33,34^.

The magnitude of N effects on litter mass remaining, C and N dynamics are also dependent on the availability of water^1^. In our study, increased precipitation amplified the positive effect of N addition on litter mass remaining and C remaining, implying that adding N can significantly suppress litter decomposition when adding water simultaneously in this ecosystem. It was found that soil fungal communities that utilize labile N sources most rapidly were closely correlated with soil moisture in alpine meadows on the Qinghai-Tibet Plateau^35^, suggesting that increased soil moisture can enhance N utilization from labile sources by soil fungi. Therefore, the amplified effects of N addition by precipitation increase may be due to that increased precipitation reduced water limitation and allow more microbial N uptake (particularly by fungi) and suppressed their lignin-degrading enzyme activity to a greater extent.

Contrary to the obvious N accumulation in the litter, P showed net losses in all the treatments. The C:P ratio in the initial litter (685.4) was much higher than the frequently found 50–150 in fungal decomposers^6^, indicating great P limitation for microbes, which has been found in other cold ecosystems (Almeida *et al*. 2018; Wang *et al*. 2017). Also, the N:P ratio in the root litter (5.8) was relatively low compared to litters in other studies^36–38^, suggesting that in the litter P was relatively less limited than N, which could have made microbes mobilize more organic P from the litter.

Although the sensitivity of decomposition to temperature is high in cold biomes such as alpine meadows^17,39^ and root decomposition has been found to be positively correlated with temperature^40,41^, we did not find any significant effects of the air warming treatment on the litter decomposition in our study. In our experiment, instead of increasing soil temperature, air warming treatment by open-top chambers (OTCs) decreased soil temperature at the depth of 10 cm (Fig. 1). It could be due to increased shading by plant aboveground growth and higher interception of radiation in the OTCs. We found that soil temperature was correlated with mass, C and N remaining of the litter irrespective of root presence or absence: all the three parameters decreased when soil temperature increased (Fig. 5). This suggests that temperature did play a role in root litter decomposition, specifically in C and N mineralization in this case, by stimulating either microbial activities or plant N uptake. However, the decrease in soil temperature was not big enough to make C and N remaining differ significantly between the two air warming treatments.

Living roots in our study showed significant effects on litter C, N, and P remaining, and root presence/absence alone accounted for 27%, 56% and 44% of the explained variance in C, N and P remaining, respectively, indicating that living roots play a critical role in litter decomposition in the Qinghai-Tibet Plateau. Living roots reduced root litter C remaining (Fig. 3a), which could be due to root priming effect that leads to more microbial enzyme production for decomposition^26^. However, N and P remaining of the litter increased when living roots were present (Fig. 3b,c). This may because of that living roots enhanced microbial activities and thus increased nutrient translocation by microorganisms such as saprophytic and mycorrhizal fungi into the litter, as fungal translocation of N has been well documented^32,33^.

In our study 1 mm mesh size litterbags were used to allow root growth in the bags. It is possible that some soil animals such as nematodes and arthropods can also enter the 1 mm mesh litterbags and influence the decomposition of the litter. Soil animals can influence litter decomposition through multiple ways. On the one hand, they can feed on soil microbes and plant roots, which might hamper litter decomposition by microbes and reduce root priming effects^42^; on the other hand, they can directly digest litter substrate and promote microbial inoculation^43^. Therefore, it is hard to estimate the net effect of soil animal on the decomposition. However, nematode and arthropod abundance in the Tibetan alpine meadow soil is relatively low compared to other ecosystems such as forests^44–46^, and the effect of soil animals may also be low in our study.

In summary, our study suggests that in future global change scenarios, the interaction between increasing precipitation and N deposition can be the major driver of early stage root decomposition in nutrient-limited ecosystems alpine meadows in the Qinghai-Tibet Plateau. Besides, soil temperature is still important in soil C and N dynamics, and if effects of climate warming is strong enough to increase soil temperature at depths, the decomposition of roots and other organic matters in the soil can be greatly influenced. Although positive relationship was found between annual precipitation and root decomposition rate^40,41^, precipitation manipulation experiments showed mixed results about precipitation change effects on root decomposition^47^. In our study precipitation reduction did not have any significant effect on measured parameters except soil moisture in our study. Some studies found that plant community and soil biotic processes were resistant to drought stresses in alpine meadows in the Qinghai-Tibet Plateau and that temperature was the main driver of the ecosystem C cycling^48,49^. Our results also suggest that microbial processes involved in litter decomposition may be resistant to moderate decreases in precipitation in alpine meadows of the Qinghai-Tibet Plateau.

## Conclusions

In our study, the decomposition of root litter in the Qinghai-Tibet Plateau was affected by N addition, increased precipitation, and living roots. N addition suppressed the litter decomposition, and this suppression was enhanced by increased precipitation. The presence of living roots significantly decreased C remaining but increased N and P content of the remaining litter. These results suggest that under future climate change scenarios, the interaction between N and precipitation may be critical for nutrient dynamics during the early stage of root litter decomposition in the Qinghai-Tibet Plateau, and that the living roots critically modulate root litter decomposition in these N-limiting alpine systems.

## Materials and Methods

### Study site

The experiment was carried out in Maqu, Gansu, China (101°53′E, 35°58′N), which is in the east of the Qinghai-Tibet Plateau, with an altitude of 3500 m. The annual mean temperature and annual precipitation of the study site were 1.8 °C and 593 mm, respectively for the period 1981–2010 from the National Meteorological Information Center. The dominant plant species were *Elymus nutans* Griseb., *Kobresia capillifolia* (Decne.) C.B.Clarke and *Stipa aliena* Keng. The soil type is Mattic Cryic Cambisols.

### Experimental design

Our field experiment was a full factorial design with manipulation of N (ambient and N addition), precipitation (ambient, precipitation reduction and increase) and temperature (ambient and air warming). Air temperatures of the plots with the air warming treatment were warmed by approximately 1–2 °C (unpublished data) through OTCs. OTCs were hexagon chambers made of 6 pieces of transparent plexiglass, with a diameter of 1.5 m at the bottom and 1.2 m at the top, and with a height of 0.5 m. On average the OTCs increased air temperature by about 1.5 °C at our site. N was added twice a year at 12 g N m^−2^ yr^−1^ in the form of urea. Precipitation was reduced by rain shelters which intercepted about 30% of ambient rainfall. For the precipitation reduction treatment, 7 v-shaped transparent plexiglasses were placed above soil surface on a metal hanger over each plot. In each plot, the transparent plexiglasses covered 30% of the soil surface area and they were tilted (the south side was 1 m above the soil surface and the north side was 1.5 m above the soil surface) so that intercepted precipitation flew to the south side, where it was collected by PVC tubes and lead to a plastic container. The water collected in one plot was then manually added into the nearest plot that was designated for precipitation increase treatment after the rainfall event ended. In this way, each plot with precipitation increase (PI) treatment received an additional 30% natural precipitation without changing the frequency of natural precipitation. Intercepted rainfall was added to plots with the PI treatment in the same block. The plots were arranged following a randomized block design with four replicates for each treatment, and in total there were 48 plots. All the treatments were initiated in 2015.

Soil temperature was monitored continuously in two of the four blocks at the depth of 10 cm during this experiment. Temperature was recorded every 30 minutes using temperature loggers (iButton DS1922L/DS1923, Maxim Integrated, US). Soil moisture was measured every minute in the other two blocks at the depth of 10 cm using soil moisture sensor (MS10, China). Soil sample (0–20 cm) of each plot was taken in July, 2017 with a soil corer (diameter 5 cm) and then shipped back to Nanjing Agricultural University, where extractable inorganic N (i.e., NO_3_^−^ and NH_4_^+^) in each soil sample was extracted with 0.5 M K_2_SO_4_ and measured on a flow injection autoanalyser (Skalar San + + CFA, Erkelenz, Germany).

To investigate effects of the presence/absence of living roots on decomposition, we used two types of litterbags with different mesh size (1 mm and 20 μm) to allow root growth or prevent root growth but allow fungal growth in the litterbags, respectively. Litterbags were made of nylon and 10 cm × 10 cm. In early May 2017, root material was collected from a typical local plant community outside the plots. Roots were washed and very thick roots were removed to make the samples more homogeneous. The diameter of roots included in this experiment was less than 3 mm. Then the roots were mixed and air-dried for 48 hours. Approximately 1.5 g of the air-dried root material was put into each litterbag. Additional 4 root samples from the air-dried root material were taken and oven-dried at 65 °C for 48 hours to measure its residual water content and initial C, N, and phosphorus (P) concentrations. Exact weight of each sample was recorded and initial dry weights were corrected for the residual water content. In each plot, we trenched a gap (1 cm wide and 20 cm long) to 15 cm deep with knife. Two litterbags with different mesh sizes were buried into the gap vertically and side by side at the same depth. There were 96 litterbags in total.

After four months, in September 2017, all litterbags were retrieved and root material (new roots grown into bags were removed) was taken out from the litterbags, washed with tap water to remove attaching soil and weighted after oven-dried at 65 °C for 48 h. All dried samples were shipped to Nanjing Agricultural University, China, where they were ground by ball mill (MM400, RETSCH, Germany) for C, N and P analysis afterwards.

### Chemical analyses

Litter C concentration was measured using the K_2_Cr_2_O_7_ oxidation method^50^. Total N concentration was analyzed using Kjeldahl apparatus (KT260, FOSS-SCINO, Denmark) by digesting approximately 200 mg ground root material in sulphuric acid and hydrogen peroxide. Total phosphorus (P) concentration was determined by colorimetric analysis (SpectraMax M5, Molecular Devices, USA) with ammonium molybdate and ascorbic acid, using the same digestion solution as the one for N concentration^51^.

### Statistical analyses

Arithmetic mean values of soil temperature and moisture were calculated by using all the monitored values in corresponding plots during four months, respectively. Nutrient (C, N, P) contents were calculated by multiplying the nutrient concentrations of each sample by the remaining mass. Mass or nutrient remaining was then reported as the percentage of initial litter mass or nutrient contents, with values greater than 100% suggesting an accumulation of the element. All the values given in this paper are arithmetic mean ± standard error. The differences in mass, nutrient remaining and C:N, C:P in litter across treatments were analyzed with linear mixed effects models with air warming, N, precipitation, mesh size as fixed factors and plot nested within block as a random effect. Pairwise relationships between litter parameters (remaining of mass, C, N and P) and soil parameters (soil temperature, moisture and inorganic N) were also checked with linear mixed models with one of the soil parameters as the fixed factor and block as a random effect. All analyses were performed with R (version 3.4.3). Linear mixed effects models were run using package *lme4* version 1.1–7^52^; *p* values were calculated by using package *lmerTest* version 2.0–20^53^; R^2^ values were calculated by using package *MuMIn* version 1.40.0^54^.

## Supplementary information

Supplementary information
