# Supplementary material for: Effects of global change factors and living roots on root litter decomposition in a Qinghai-Tibet alpine meadow
Source: Sci Rep. 2019 Nov 15;9:16924. doi: 10.1038/s41598-019-53450-5 (PMC6858338; doi:10.1038/s41598-019-53450-5)
Supplement: Supplementary file 1 — Supplementary information [file 41598_2019_53450_MOESM1_ESM.docx]

**Supplementary information**

**Effects of global change factors and living roots on root litter decomposition in a Qinghai-Tibet alpine meadow**

Meng Shu^1^, Qingzhou Zhao^1^, Zhen Li^1^, Lin Zhang^1^, Shuijin Hu^1,2^, Peng Wang^1,^*

**Complete affiliations:**

^1^Colloge of Resources and Environmental Sciences, Nanjing Agricultural University, Nanjing, Jiangsu 210095, China

^2^Department of Entomology &Plant Pathology, North Carolina State University, Raleigh, NC 27695, USA

**Corresponding author:**

Peng Wang

College of Resources and Environmental Sciences

Nanjing Agricultural University

Nanjing, Jiangsu, 210095, China

Phone: +86(25) 84399827

E-mail: [peng.wang@njau.edu.cn](mailto:peng.wang@njau.edu.cn)

**Table S1.** Summary of ANOVA analyses of nitrogen addition (N), air warming (W) and precipitation alteration (P) on inorganic N, soil temperature and soil moisture. Significant effects (*p* < 0.05) are marked in bold.

| Source of variance | Inorganic N | | |  | Temperature | | |  | Moisture | | |
| --- | --- | --- | --- | --- | --- | --- | --- | --- | --- | --- | --- |
|  | df | *F* | *p* |  | df | *F* | *p* |  | df | *F* | *p* |
| N | 1 | 66.684 | **<0.001** |  | 1 | 52.091 | **<0.001** |  | 1 | 0.572 | 0.465 |
| W | 1 | 12.487 | **0.001** |  | 1 | 40.122 | **<0.001** |  | 1 | 5.917 | **0.033** |
| P | 2 | 0.812 | 0.452 |  | 2 | 1.572 | 0.247 |  | 2 | 4.121 | **0.046** |
| N×W | 1 | 6.395 | **0.016** |  | 1 | 3.703 | 0.078 |  | 1 | 0.704 | 0.419 |
| N×P | 2 | 3.046 | 0.061 |  | 2 | 0.882 | 0.439 |  | 2 | 0.675 | 0.528 |
| W×P | 2 | 0.023 | 0.977 |  | 2 | 0.542 | 0.595 |  | 2 | 0.557 | 0.587 |
| N×W×P | 2 | 0.454 | 0.639 |  | 2 | 0.038 | 0.962 |  | 2 | 1.463 | 0.273 |

**Table S2.** Soil characteristics of different treatments used in the study. Values given are arithmetic mean ± standard error (*n* = 2 for soil temperature and moisture; *n* = 4 for inorganic nitrogen). CT, control; W, air warming; N, nitrogen addition; PI, precipitation increase; PR, precipitation reduction.

| Treatment | CT | PI | PR | W | N | N×PI | N×PR | W×PI | W×PR | N×W | N×W×PI | N×W×PR |
| --- | --- | --- | --- | --- | --- | --- | --- | --- | --- | --- | --- | --- |
| Soil temperature (°C) | 14.82±0.43 | 15.15±0.23 | 14.80±0.33 | 13.48±0.06 | 13.67±0.09 | 13.71±0.17 | 13.68±0.37 | 14.11±0.3 | 13.82±0.13 | 12.91±0.24 | 13.09±0.12 | 13.26±0.04 |
| Soil moisture (%) | 24.00±0.26 | 26.37±4.17 | 24.69±1.00 | 23.68±0.45 | 24.02±3.39 | 24.08±0.10 | 18.59±4.47 | 21.40±2.11 | 18.40±2.28 | 21.36±1.15 | 22.76±0.34 | 19.65±1.63 |
| Inorganic nitrogen (mg/kg) | 5.48±0.82 | 6.50±0.46 | 3.78±1.01 | 4.33±0.69 | 20.31±6.92 | 17.56±5.11 | 29.99±4.41 | 14.04±7.05 | 6.67±1.03 | 42.06±3.93 | 33.64±5.47 | 48.17±14.62 |

**Table S3.** Summary of ANOVA analyses of nitrogen addition (N), air warming (W), precipitation alteration (P) and root presence/absence on the decomposition of root litter. Significant effects (*p* < 0.05) are marked in bold.

| Source of variance | Mass remaining | | |  | C remaining | | |  | N remaining | | |  | P remaining | | |  | C:N | | |  | C:P | | |
| --- | --- | --- | --- | --- | --- | --- | --- | --- | --- | --- | --- | --- | --- | --- | --- | --- | --- | --- | --- | --- | --- | --- | --- |
|  | *df* | *F* | *p* |  | *df* | *F* | *p* |  | *df* | *F* | *p* |  | *df* | *F* | *p* |  | *df* | *F* | *p* |  | *df* | *F* | *p* |
| N | 1 | 3.941 | 0.055 |  | 1 | 4.025 | 0.053 |  | 1 | 9.831 | **0.003** |  | 1 | 0.107 | 0.744 |  | 1 | 6.043 | **0.016** |  | 1 | 0.144 | 0.705 |
| W | 1 | 0.496 | 0.486 |  | 1 | 0.403 | 0.529 |  | 1 | 0.028 | 0.867 |  | 1 | 2.117 | 0.154 |  | 1 | 0.096 | 0.758 |  | 1 | 2.117 | 0.154 |
| P | 2 | 0.969 | 0.390 |  | 2 | 0.793 | 0.460 |  | 2 | 0.865 | 0.426 |  | 2 | 0.333 | 0.718 |  | 2 | 0.668 | 0.516 |  | 2 | 0.083 | 0.920 |
| Root | 1 | 0.722 | 0.401 |  | 1 | 5.412 | **0.026** |  | 1 | 25.640 | **<0.001** |  | 1 | 7.781 | **0.008** |  | 1 | 33.006 | **<0.001** |  | 1 | 8.487 | **0.006** |
| N×W | 1 | 1.191 | 0.283 |  | 1 | 0.285 | 0.597 |  | 1 | 0.781 | 0.380 |  | 1 | 0.191 | 0.664 |  | 1 | 1.360 | 0.248 |  | 1 | 0.131 | 0.718 |
| N×P | 2 | 3.571 | **0.039** |  | 2 | 3.060 | 0.060 |  | 2 | 3.001 | 0.056 |  | 2 | 2.047 | 0.143 |  | 2 | 1.571 | 0.215 |  | 2 | 2.111 | 0.135 |
| W×P | 2 | 1.310 | 0.283 |  | 2 | 1.418 | 0.256 |  | 2 | 0.901 | 0.411 |  | 2 | 0.42 | 0.660 |  | 2 | 1.573 | 0.215 |  | 2 | 0.086 | 0.917 |
| N×Root | 1 | 0.185 | 0.670 |  | 1 | 0.274 | 0.603 |  | 1 | 0.183 | 0.670 |  | 1 | 0.037 | 0.848 |  | 1 | 0.328 | 0.568 |  | 1 | 0.009 | 0.924 |
| W×Root | 1 | 0.034 | 0.856 |  | 1 | 0.024 | 0.876 |  | 1 | 0.509 | 0.478 |  | 1 | 0.075 | 0.784 |  | 1 | 0.315 | 0.577 |  | 1 | 0.466 | 0.499 |
| P×Root | 2 | 1.567 | 0.223 |  | 2 | 1.418 | 0.255 |  | 2 | 0.659 | 0.521 |  | 2 | 0.577 | 0.566 |  | 2 | 0.887 | 0.416 |  | 2 | 0.690 | 0.507 |
| N×W×P | 2 | 0.721 | 0.494 |  | 2 | 0.678 | 0.514 |  | 2 | 1.758 | 0.180 |  | 2 | 0.268 | 0.765 |  | 2 | 0.995 | 0.375 |  | 2 | 0.531 | 0.592 |
| N×W×Root | 1 | 0.400 | 0.531 |  | 1 | 0.013 | 0.908 |  | 1 | 0.098 | 0.755 |  | 1 | 0.012 | 0.912 |  | 1 | 0.069 | 0.794 |  | 1 | 0.053 | 0.817 |
| N×P×Root | 2 | 0.426 | 0.657 |  | 2 | 1.864 | 0.169 |  | 2 | 0.777 | 0.464 |  | 2 | 1.783 | 0.182 |  | 2 | 1.539 | 0.222 |  | 2 | 1.755 | 0.187 |
| W×P×Root | 2 | 0.003 | 0.997 |  | 2 | 0.504 | 0.607 |  | 2 | 0.149 | 0.862 |  | 2 | 0.647 | 0.529 |  | 2 | 0.300 | 0.741 |  | 2 | 1.075 | 0.351 |
| N×W×P×Root | 2 | 0.034 | 0.966 |  | 2 | 0.054 | 0.947 |  | 2 | 0.413 | 0.663 |  | 2 | 1.268 | 0.293 |  | 2 | 0.444 | 0.643 |  | 2 | 1.401 | 0.259 |


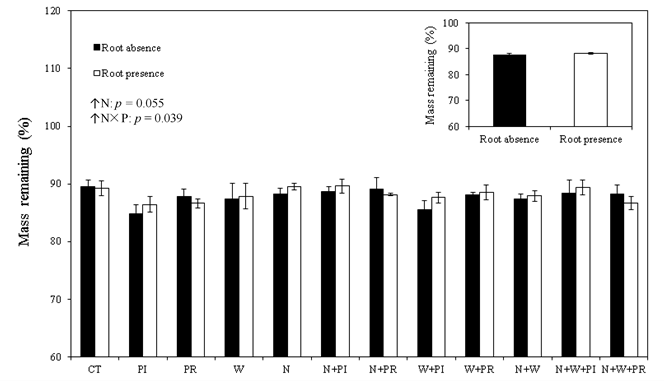


**Figure S1** Mass remaining of the litter of mixed roots from dominant species on the Qinghai-Tibet Plateau in different treatments. CT, control; W, warming; N, nitrogen addition; P, precipitation condition; PI, precipitation increase; PR, precipitation reduction. All datawere expressed as % of original litter mass.Bars show mean ± SE (*n* = 4).


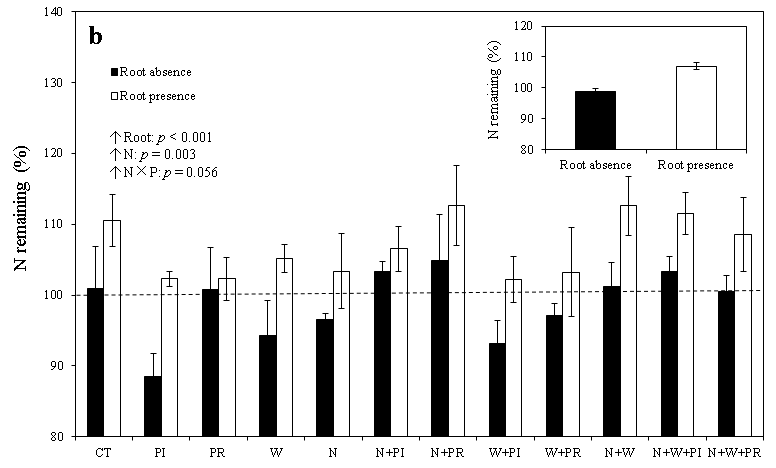

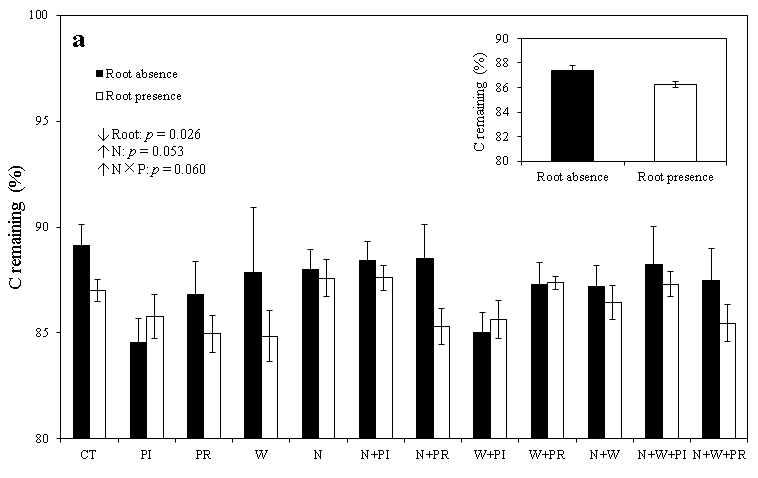


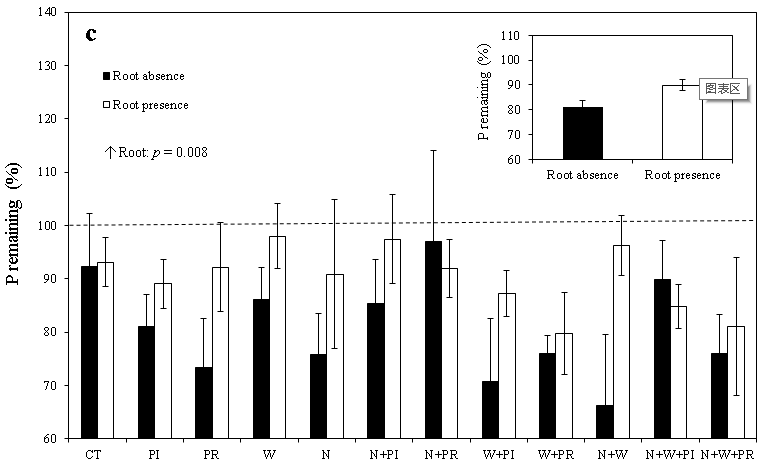


**Figure S2** C (a), N (b) and P (c) remaining of the litter of mixed roots from dominant species on the Qinghai-Tibet Plateau in different treatments. CT, control; W, warming; N, nitrogen addition; P, precipitation condition; PI, precipitation increase; PR, precipitation reduction. All datawere expressed as % of original litter C, Nand P content.Bars show mean ± SE (*n* = 4).
